# Molecular phylogenetics of a recently isolated goat pox virus from Vietnam

**DOI:** 10.1186/s12917-021-02777-1

**Published:** 2021-03-08

**Authors:** Trang Hong Pham, Nor Yasmin Abdul Rahaman, Mohd Azmi Mohd Lila, Huong Lan Thi Lai, Lan Thi Nguyen, Giap Van Nguyen, Bo Xuan Ha, Hieu Nguyen, Hanh Duc Vu, Mustapha M. Noordin

**Affiliations:** 1 Faculty of Veterinary Medicine, Universiti Putra Malaysia, 43400 Serdang, Selangor Malaysia; 2 Faculty of Veterinary Medicine, Hanoi University of Agriculture, Gia-Lam District, Hanoi, 10000 Vietnam; 3 Faculty of Animal Science, Hanoi University of Agriculture, Gia-Lam District, Hanoi, 10000 Vietnam; 4 National Institute for Control of Vaccine and Biologicals, Ministry of Health, Hoang-Mai District, Hanoi, 10000 Vietnam

## Abstract

**Background:**

After a decade of silence, an outbreak of the contagious and Asian endemic disease, goat pox re-emerged in North Vietnam affecting more than 1800 heads with a mortality rate of 6.5%. The inevitable impact of goat pox on hide quality, breeding, chevon and milk production has resulted in a significant economic losses to the developing goat industry of Vietnam. In the act of establishing an effective control of this devastating disease, tracing the source of re-emergence via a phylogenetic study was carried out to reveal their genetic relatedness. Either skin scab or papule from the six affected provinces were collected, cultured into Vero cells followed by restricted enzyme digestion of targeted P32 gene DNA encoding. The P32 gene was then cloned and transformed into *E.coli* competent cells for further sequencing.

**Results:**

The isolated sequence is deposited into GenBank under Accession No. MN317561/VNUAGTP1. The phylogenetic tree revealed high similarity of nucleotide and amino acid sequences to references goat pox strains accounting for 99.6 and 99.3, respectively. The Vietnamese strain is clustered together with currently circulating goat pox virus in China, India and Pakistan which suggested the origin of South China.

**Conclusions:**

This Vietnam isolate is clustered together with other Asian goat pox strains indicating the dissemination of a common goat pox virus within this continent.

## Background

Goat pox is a highly infectious disease of goats that is prevalent in Asia, Middle-East and Africa caused by the enveloped double stranded DNA virus from the Poxviridae [1]. The disease is transmitted by direct (aerosol and nasal secretions) or indirect contact (contaminated object and mechanically by insect) [2]. Clinically, affected goats developed fever, generalised cutaneous papules or nodules which may disseminate to the respiratory and gastrointestinal systems [3].

There is a dearth of documentation on goat pox in Vietnam with an earliest report only in 2005. During that year, the Department of Animal Health, Vietnam reported a nationwide outbreak with a 76.39% case fatality rate. Later, analysis showed that the isolate originated from China [1]. This connection is expected since capripox infection that frequently occurs in China may spill over into Vietnam’s which is China immediate southern neighbour [4]. This poses an economic threat to Vietnam’s infant caprine industry, despite successfully registering a goat population increase from 1.3 to 2.6 million heads (2013–2018). Therefore, a comprehensive analysis and mapping of Vietnam goat pox outbreak is warranted for an effective control campaign including a yielding a potential vaccine.

Genetic analysis techniques allow an investigation of capripox at genera level purportedly for diagnostic and prevalence studies. Targeting gene of P32, GPCR (G-protein-coupled chemokine-receptor) and RPO30 (30 kDa DNA-dependent RNA polymerase subunit) are commonly asserted for capripoxviruses differentiation [5–9]. However, as carried out in this study, only P32 gene-based PCR-RFLP is employed which is sufficient to unveil similarities to a nucleotide and amino acid sequences of goat pox strains [5, 6, 10–12].

Nevertheless, the eruption of outbreaks of goat pox in vaccinated animals explains its abandonment in Europe [13–15]. This signals the possibility of an outbreak to originate from vaccine strains too. A successful control and prevention goat pox outbreak can be achieved if the causal strain is known. Thus, this study of the tracing the strains sparking the recent goat pox outbreak in Vietnam is of utmost importance in abating the spread of this disease.

## Methods

Specific methodologies are described below while the generic methodology including the experimental design and ethical clearance are as previously published [4].

### Samples

Samples for the current study were collected based on the epidemiology data that was previously reported [4]. A total of 128 skin scab samples placed in PBS were obtained from suspected goats in North Vietnam encompassing Bac Giang (BG1), Ha Noi (HN1), Hoa Binh (HB1), Nghe An (NA1), Ninh Binh (NB1 and NB2) and Yen Bai (YB1). Vaccine strain was derived from a live attenuated vaccine (titer of at least 10^3.5^ TCID_50_, GTQ strain) by NAVETCO (Ho Chi Minh City, Vietnam; National Assigned No. TW-XI-85) and used as a positive control.

### Polymerase chain reaction (PCR)

The collected skin scab samples were subjected to the standard virology procedure as documented by OIE [16]. A 10% of virus suspension was prepared in PBS (Tablets, USA) with the addition of 1% antibiotics (Gibco, USA) and centrifuged at 3500 rpm for 10 min. The supernatant was then collected through 0.45 μm nitrocellulose membrane filter (Minisart Syringe Filter, USA) and stored at -80 °C. DNA extraction from prepared cell suspension were performed using QIAamp DNA Mini Kit (Qiagen, Germany) as described by manufacturer. The eluted DNA was stored at -20 °C until further use.

The PCR reaction was carried for Capripox virus genome using primary primer set designed by Ireland and Binepal [17], i.e. P1F (5′-TTTCCTGATTTTTCTTACTAT-3′) and P2R (5′-AAATTATATACGTAAATAAC-3′). The PCR reagents included 12 μL of template DNA, 2 μL of 10 mM dNTP, 2 μL of 10 pmol/ml of each primer, 0.4 μL of Taq DNA polymerase, 5 μL PBS, 3 μL 2 mM MgCl_2_ with the addition of nuclear free water to make total volume of 50 μL. The thermo-cycle condition was started with 5 min of initial denaturation at 94 °C, followed by 35 cycles of denaturation at 94 °C in 1 min, annealing at 50 °C in 1 min and extension at 72 °C in 1 min. The final extension was performed at 72 °C for 10 min then later preserved at 4 °C.

The PCR procedure using P1F–P2R was performed at fifth, seventh and ninth passages following the appearance of CPE. Stabilised adaptation virus strains at 5 days PI of the ninth passage were collected and stored at -80 °C for further usage.

### Virus isolation

Cell culture was performed using Vero cells as recommended by the OIE protocol [18]. Briefly, 102 positive capripox virus PCR samples were inoculated to a 25 cm^3^ cell culture flask (Corning, Mediatech Inc., Virginia, USA) of 80% confluent monolayer for penetration at 37 °C for 2 h, then washed thrice where maintenance medium was added with 2% foetal calf serum (Corning, Corning, Mediatech Inc., Virginia, USA) and 1% antibiotics (Gibco, USA). The cultures were incubated at 37 °C with 5%CO_2_ with frequent changing of medium every 2 days. Cytopathic effect (CPE) was recorded daily under an inverted microscopic (Zeiss Axiovert 4 °C, Germany) for 14 days. Negative culture was considered when there was absence of CPE following two or more blind passages.

### Amplification of P32 gene

Extracted DNA that was previously positive from initial P1F-P2R primers reaction and produced above 80% of CPE in Vero cells was utilized for a second PCR procedure (95 samples). The specific primers for P32 gene were designed by National Center of Veterinary Diagnosis, Vietnam which were EP32F (5′-CCCGAATTCATGGCAGATATCCCATTATATG-3′) and EP32R (5′-CCGAAGCTTCTAACTATATACGTAAATAAC-3′) which amplify 969 bp fragment of P32 gene. Total 50 μL PCR reaction contained 25 μL PCR master mix (Fermentas, USA), 2 μL 10 pmol/ml of each primers, 2.5 μL DMSO, 2 μL template DNA and 16.5 μL nuclease free water. Thermal cycles were as follows: initial denaturation at 94 °C for 5 min, 35 cycles of denaturation at 94 °C for 1 min, annealing at 55 °C for 1 min, extension at 72 °C for 1 min. Then, the final extension was at 72 °C for 10 min which later preserved at 4 °C.

The DNA length of isolated strains was measured using 1.5% agarose gel electrophoresis with EtBr staining while the minimum amount of DNA eluted onto the agarose gel was 10 ng. The DNA fragment was visualized using UV light device (Analytikjena, USA) and band size was defined based on manufacturer’s DNA ladder. The PCR-positive product was purified using QIAquick PCR Purification Kit (QIAGEN Inc., USA) following the producer’s procedure. The purified DNA was then stored at -20 °C until further cloning.

Amplification using the P32 specific primer pairs (EP32F-R) was conducted on above DNA templates (five studied virus strains and three field isolates). Gel electrophoresis captured a clear DNA band of PCR products, about 1 kb in size (Fig. 3). The result showed that the PCR reaction yielded only a high quality single product of 969 bp.

The purified PCR products using QIAquick PCR Purification Kit (QIAGEN Inc.) was cloned in pCR™2.1-TOPO® vector (TA-cloning Kit. Invitrogen), later transferred into One Shot® DH5α™-T1®. The transformed competent cell was then cultured on agar and positive colony (white color) was used for isolating plasmid DNA using QIAprep Spin Miniprep Kit. The addition of *Eco*RI restriction enzyme into positive suspension for verification of plasmid DNA where 1% agarose electrophoresis is shown both DNA vector and inserted bands (Fig. 4).

### Plasmid cloning and transformation in competent cells

In present study, TOPO® TA Cloning Kits (Invitrogen, USA) with pCR™2.1-TOPO® vector and One Shot® DH5α™-T1® (chemically *E.coli* competent cells) was employed. Briefly, TOPO® TA Cloning was prepared according to the manufacturer’s instruction with addition of PCR products, then the mixture of TOPO® cloning reaction and the prepared One Shot® chemically competent *E. coli* was incubated overnight on X-gal LB plate at room temperature.

### Isolation and confirmation of plasmid DNA

Plasmid DNA was isolated from positive clones (white or light blue) using QIAprep Spin miniprep Kit (QIAgen, USA) as described by manufacturer. In order to fully confirmed usage of positive clones, the plasmid DNA was verified using restriction enzyme by incubating only the white clones suspension with enzyme *Eco*RI at 37 °C for 2 h. The DNA was checked by using 1% agarose. The positive band was represented by both DNA vector and inserted bands.

### Sequencing and phylogenetic analysis

Since the obtained five DNA plasmids yielded identical sequence, sequencing was conducted on one positive plasmid DNA using specificity primers M13F (5′-GTAAAACGACGGCCAG-3′) and M13R (5′-CAGGAAACAGCTATGAC-3′). The selected product was purified using DyeEx 2.0 Spin Kit (QIAGEN, USA) before sequencing. The purified product was then freeze-dried and automatically assembled and consensus level sequences generated in ABI-3100 Avant Genetic Analyzer (ABI PRISM, USA). The strain sequence was deposited into GenBank under Accession No. MN317561/VNUAGTP1.

The phylogenetic analysis was carried out using DnaSP software [19] while MEGA software [20] that was used to construct & infer the phylogenetic tree. Neighbour-joining trees with 1000 bootstrap [21] replicates were generated and Tamura-3P model [22]. was used to emulate nucleotide transformation. Reference sequences for use in analysis alongside sequencing generated in this study was downloaded from the NCBI as shown in Table 1.

**Table 1:** The comparative analysis of nucleotide and amino acid sequences of P32 gene

No.	Accession No.	Country of Origin	Species	Nucleotide	Amino acid
1	MN317561	Vietnam	GTPV	100	100
2	MG817382	China	GTPV	99.89	99.69
3	MG458381	China	GTPV	99.79	99.69
4	MK507852	Pakistan	GTPV	99.79	99.69
5	KY389314	India	GTPV	99.79	100
6	KP702291	India	GTPV	99.79	99.69
7	KF468757	India	GTPV	99.89	100
8	MG188745	India	GTPV	99.69	99.69
9	EF522179	China	GTPV	99.79	99.38
10	MH545960	India	GTPV	99.69	99.38
11	KX576657	Iran	GTPV	99.59	99.69
12	AY077835	Kazakhstan	GTPV	99.48	99.69
13	EF522181	China	GTPV	99.28	99.38
14	MG458383	China	GTPV	99.07	98.76
15	MN072624	Sudan	GTPV	99.17	99.69
16	MN072625	Yemen	GTPV	99.07	99.69
17	MN072623	Oman	GTPV	98.97	99.38
18	MN072619	Kenya	LSDV	98.86	97.52
19	KY829023	Greece	LSDV	98.86	97.52
20	KY702007	Serbia	LSDV	98.86	97.52
21	KX683219	Kenya	LSDV	98.86	97.52
22	MK607130	India	SPPV	98.04	96.90
23	MG458369	China	SPPV	98.04	97.21
24	MH924594	Tunisia	SPPV	98.04	96.90
25	MH198040	India	SPPV	98.04	96.90

## Results

### PCR

Out of the 128 collected samples, 102 were confirmed to be positive for capripox virus which as then cultured. Out of these, 95 samples have induced stabilised CPE that was later selected for the second PCR amplification of P32 gene.

**Fig. 1:**
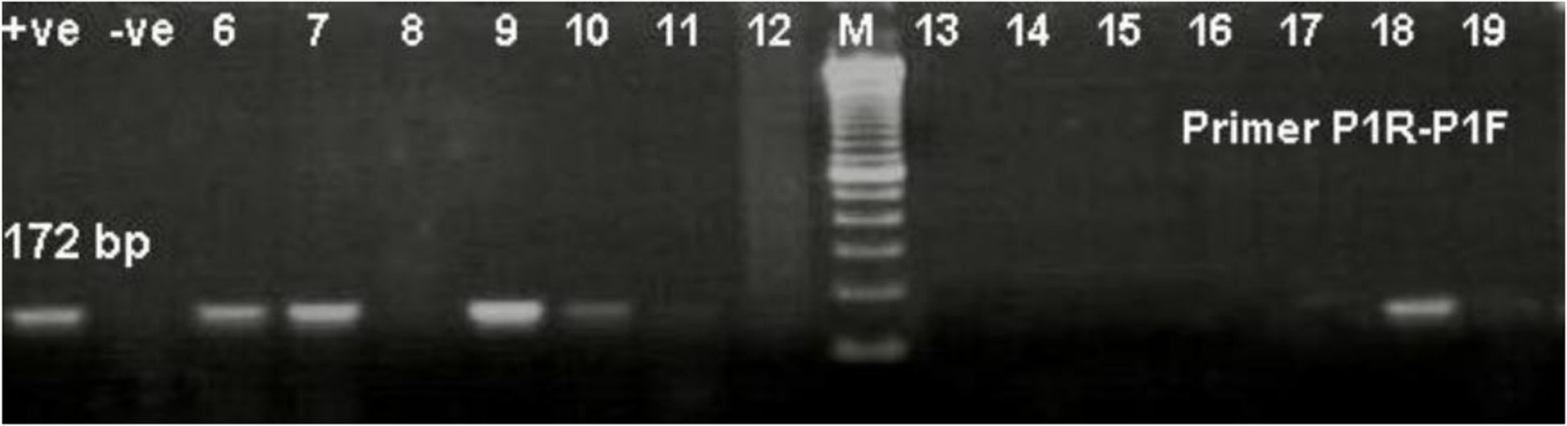
Confirmation of Capripoxvirus using PCR (Primer P1R-P1F) amplifying 172 bp of positive field isolated strains (lane 6, 7, 9, 18). +ve: vaccine strain. M: 100 bp ladder

Figure 1 shows the polyacrylamide gel electrophoresis (PAGE) analyses of PCR products from the tested scabs. The P1(F-R) specific primers for pan capripox virus PCR was used to determine the presence of capripox virus genome using 1% agarose electrophoresis. All suspected samples expressed single amplicon of aproximately 172 bp of the virus isolated upon inoculation onto Vero cells where out of the 128 samples tested, 102 were positive.

### Virus isolation

The time taken for cytopathic effect (CPE induction by the five strains, viz.; NB1, HN1, NA1, YB1, HB1) of goat pox virus on Vero cells was seen as early at 8 days post-infection (PI) while the other strains (BG1, NB2) attained CPE the following day. Following the second and third passages, six strains induced CPE at 7 days PI. The appearance of CPE was reduced to 6 days PI with the fourth passage onwards. By the seventh passage, all isolates showed adaptation on Vero cells with shortest interval for CPE formation occurring at 5 days PI.

**Fig. 2:**
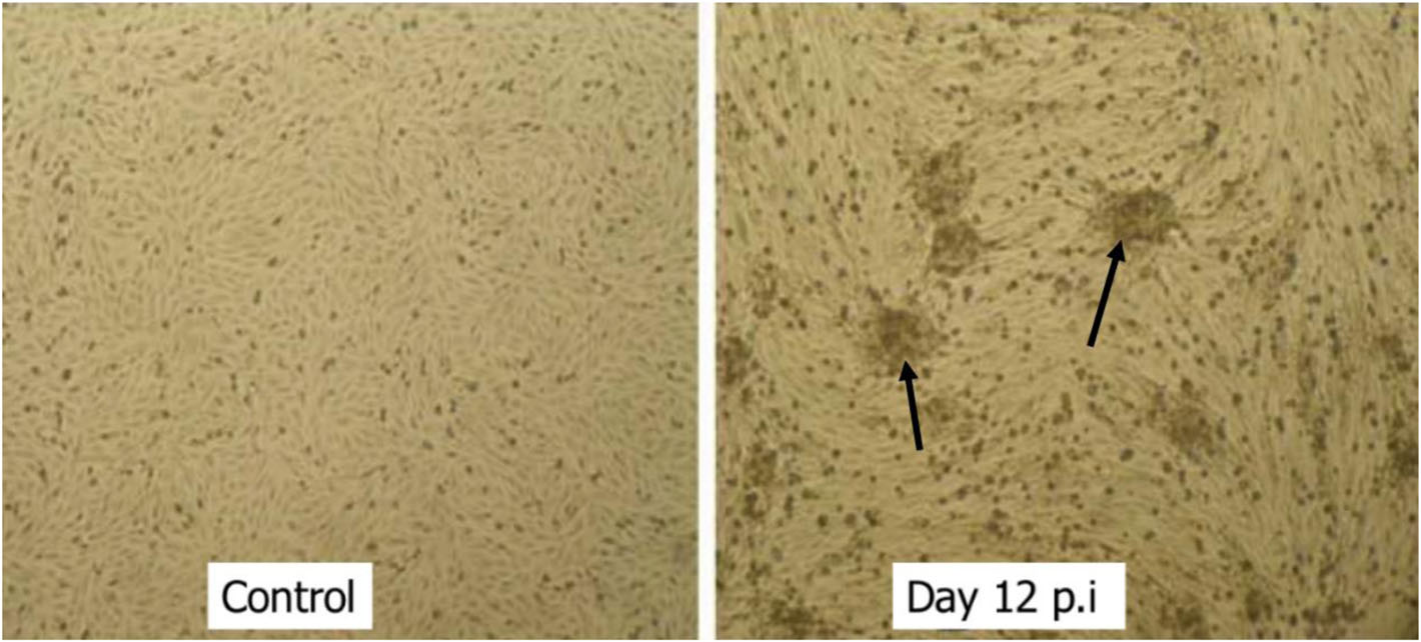
Spindle-shaped cells of negative control Vero cell and characteristic CPE of aggregation (arrows) at 12 DPI with Vietnamese goat pox strain (unstained, 100X)

The cells of the negative control were slightly round or spindle shaped forming a monolayer confluent cell sheet (Fig. 2). The appearance of the first CPE at 8 days PI was characterized by cytoplasmic granulation of infected cells rounding-off and aggregated forming clustered or clumped cells. Commencing from 10 days PI onwards, the CPE gradually increased and reached more than 80% detachment. In subsequent (second passage onward) passages, although a similar CPE appearance was recorded earlier (5 days PI), the formation of syncytium and detachment was quicker as early as 48 h.

### P32 gene amplification

**Fig. 3:**
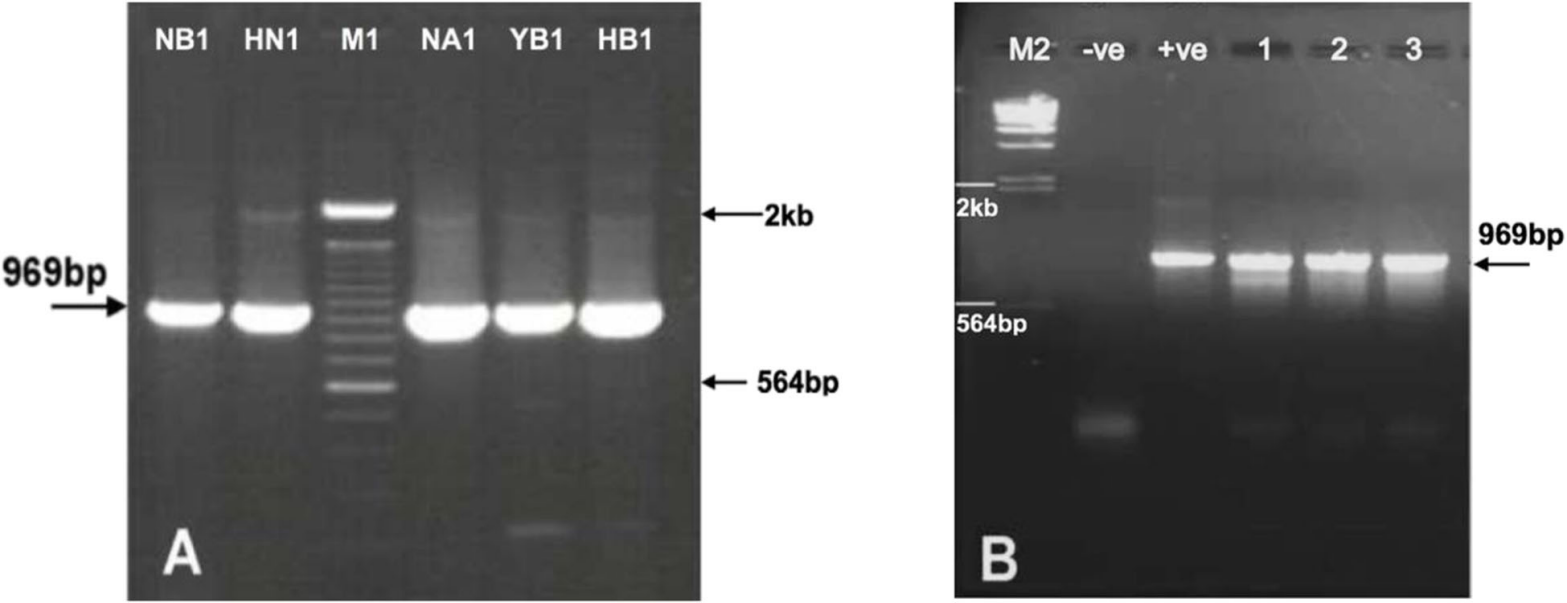
The electrophoresis results of PCR products revealed the P32 gene of 969 bp length from five GTPV strains (A); and 3 GTPV field isolates (B, lane 1 to 3); Positive control (+ve) and negative control (-ve); M1 and M2: 100 bp DNA ladder

**Fig. 4:**
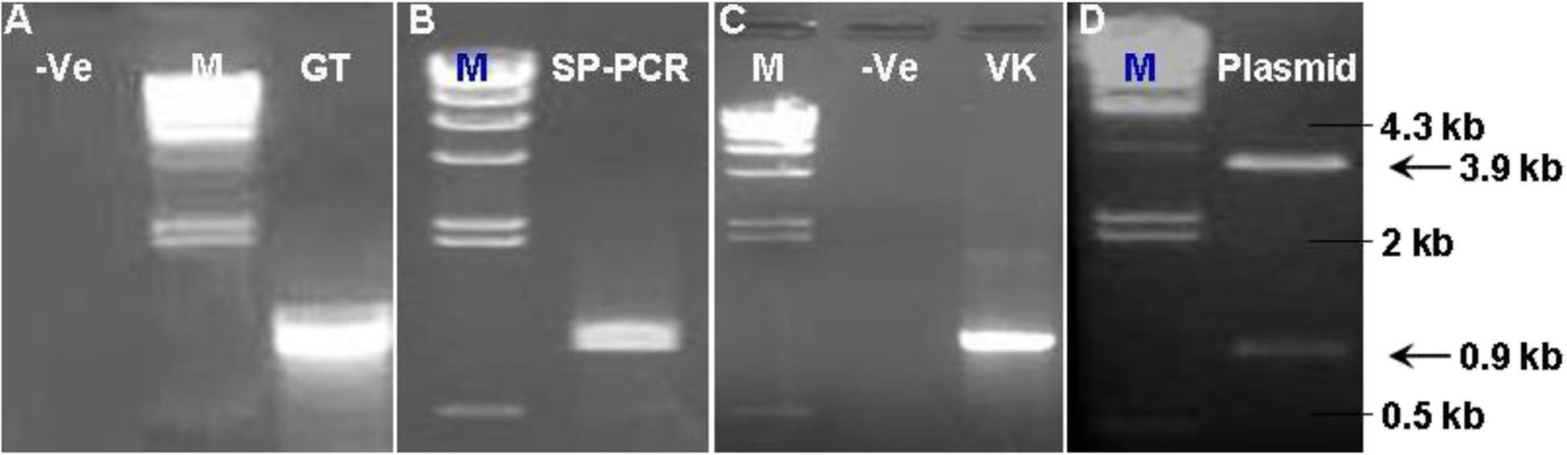
Plasmid cloning and confirmation of the plasmid DNA. The PCR using specific primers EP32(F-R) for P32 gene. -ve is negative control of uninfected goat primary testicular culture; Positive band of 0.9 - 1 kb using viral infected goat primary testicular cells (A); Purification PCR product (SP-PCR) showed a clear 0.9 kb single band (B); The ligated PCR product in vector and transformed into competent E.coli (VK, 0.9 kb) (C); Representative of positive colony was screened for confirmation of plasmid DNA (3.9 kb - vector) and inserted band (0.9 kb - P32 gene) (D); M: 100 bp DNA ladder

The gel electrophoresis captured a clear DNA band of PCR products, about 1 kb in size (Fig. 3). The result showed that the PCR reaction yielded only a high quality single product of 969 bp. The addition of *Eco*RI restriction enzyme into positive suspension for verification of plasmid DNA is shown in Fig. 4.

The samples revealed a P32 gene with fragment of 969 bp length and encoded for 323 amino acids. The nucleotide analysis result showed a similarity score to other goatpox virus strains ranging from 98.97–99.89%. A closer association (homogeneity rate of 98.86 and 98.04%, respectively) was seen in this isolated Vietnam goat pox virus to lumpy skin disease virus (LSDV) than that of sheep pox virus (SPPV).

The analysis of the amino acid sequence of this isolated Vietnam goat pox virus strain showed a complete homology to other goat pox virus strains generated from the GenBank with homogeneity percentages ranging from 99.38–100%.

### Analysis of nucleotide and amino acid sequences of P32 gene

The gene fragment encoding P32 protein of Capripoxviruses ranged from 966, 969 to 972 nucleotide. This change reflected a number of strains with extra nucleotide mutations: there are two goat pox virus strains carrying six nucleotide mutations (EF522181 and MG458383) and three SPPV strains carrying three extra mutations (MG458369, MH924594 and MH198040). The analysis of results using DnaSP software [17] showed that the gene encoding the P32 protein has 925 invariable sites, accounting for 95.76% homology.

A total of 41 (4.24%) sites had mutations (polymorphic nucleotide). Of these positions, 27 are classified as “information-site” (parsimony-informative site) where there are at least two changes in nucleotide type and each type of nucleotide change occurs at least two different sequences, including 77, 78, 144, 153, 192, 284, 300, 324, 330, 336, 401, 407, 412, 531, 561, 588, 639, 654, 660, 663, 670, 678, 831, 876, 919, 924 and 973. Nucleotide alterations resulting in a different coded amino acid change between goat pox, sheep pox (SPPV) and lumpy skin disease (LSDV) mainly occurred at nucleotide site of the first or the 2nd of the triple encoder.

The acid amino sequence of capripox P32 protein from the present study and the GenBank reference were analyzed. The comparison of amino acid sequence yielded that three species of Capripoxvirus genus bears similarities ranging from 95.6 to 99.3%. The amino acid positions that differ between goat pox, SPPV and LSDV are: S25D, V95A, Y138H, M292I and V325I. Notably, the Chinese goat pox vaccine strain (EF522181) had an additional two amino acid KK at position 33 and 34 compared to the remaining strains. There are different change in amino acid sequence which featured differentiative of LSDV (L52P and N306D) and SPPV (addition of aspartic acid at position 55 - resulted from the mutation of three nucleotides, P64L, L134S and T136I).

### Phylogenetic relationship of Vietnam isolated goat pox virus strain

**Fig. 5:**
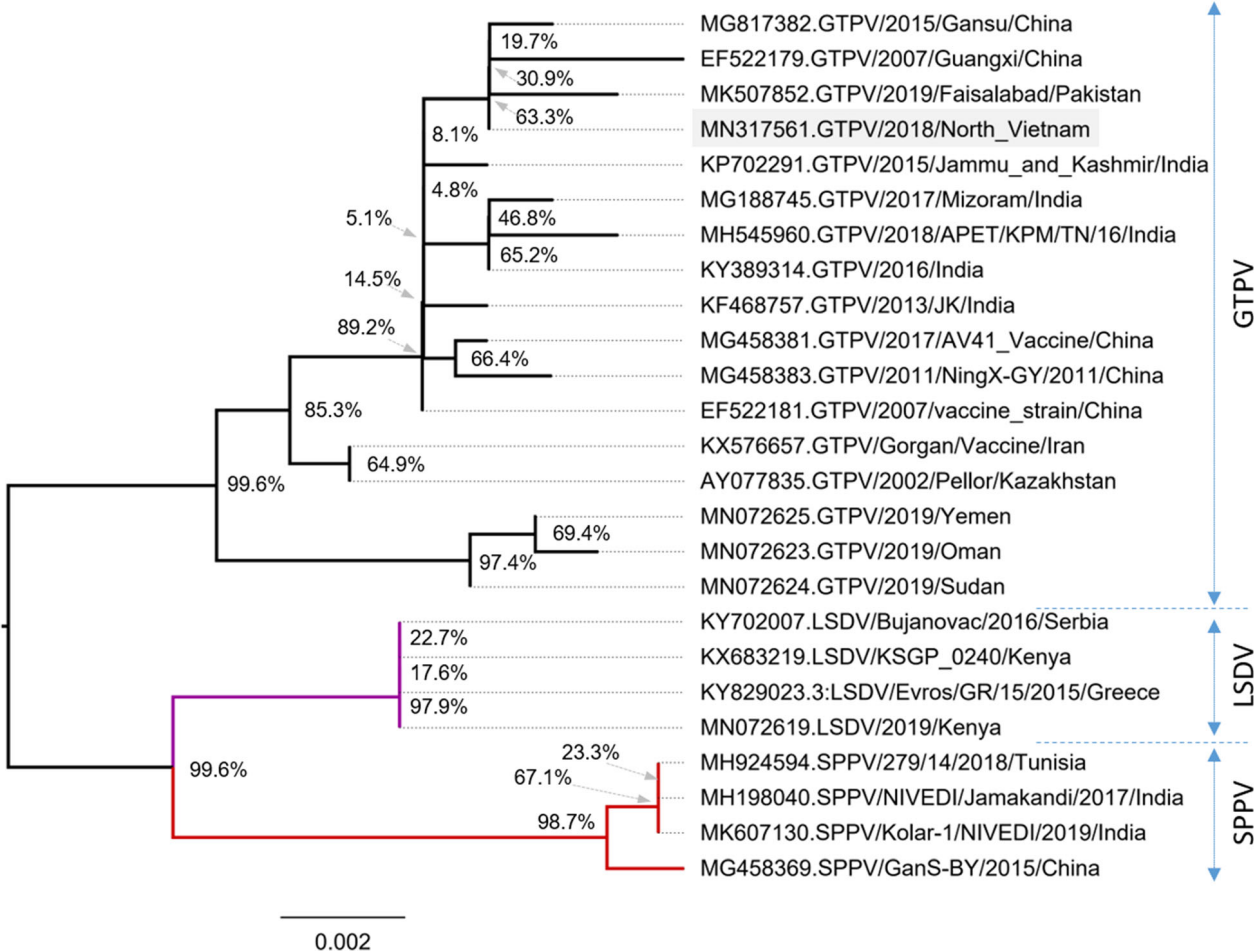
Phylogenetic analysis of different capripoxviruses based on the nucleotide sequences of P32 protein. The goat pox virus strain isolated in the North Vietnam is highlighted in grey

Based on nucleotide sequence of P32 gene, the viruses of genus capripoxvirus were divided into three distinct groups with highly reliability of branching bootstrap values (99.6%; Fig. 5). With current database, the goat pox strain of Vietnam is within the same group to that with the Pakistan (MK507852) and China (MG817382, EF522179) strains. Based on the sequence of genes encoding the P32 protein, there was no clear branching between the vaccine and goat pox virus strains.

**Fig. 6:**
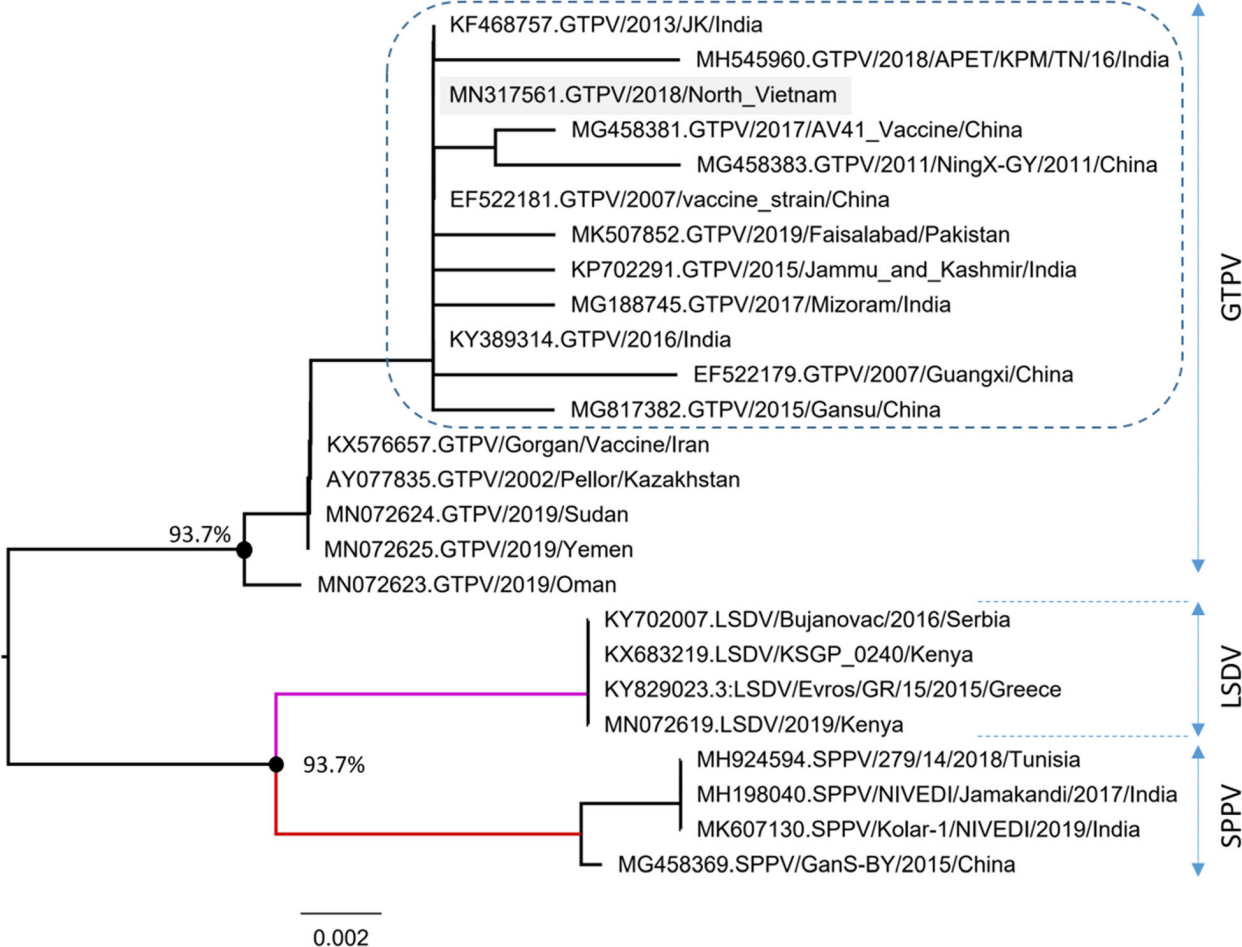
Phylogenetic analysis of different capripoxviruses based on amino acid sequence of P32 gene. The present studied goat pox strain is clustered together with strains currently circulating in India, China and Pakistan

Building a phylogenetic tree based on amino acid sequence of P32 gene rendered similar results (Fig. 6). Specifically, three member of the Capripoxviruses including goat pox, SPPV and LSDV were tested. Goat pox virus, SPPV and LSDV were observed with high branching values (93.7%). Based on branching characteristics, the goat pox strain isolated in the North Vietnam is clustered together with goat pox strains circulating in India, China and Pakistan (dashed area).

## Discussion

Chronologically, in March 2014, the first re-emergence of goatpox outbreak was reported in Yen Son district, Ninh Binh Province. A total of 371 goats out of a population of 749 were affected with a mortality rate of almost 92%. Signs seen in these goats were typical nodules on the face and other thin or hairless skin of abdomen including the vulva [4]. Despite restricted movement of goats imposed by the Vietnam Department of Animal Health, cases continue to happen until the early 2015. It has stretched over the entire province and extended into Bac Giang, Ha Nam, Ninh Binh provinces. However, the outbreak in Yen Bai province (closer to the Vietnam-China border) was due to the introduction of 50 goats from China.

Thus, illegal trafficking of goats across the border along with movement of goats those from affected farms and borders were believed as contributors to the outbreak. The permeable nature of the border between Vietnam and China has heighten the transmission of goatpox. In addition, in North Vietnam provinces bordering China, it is inevitable for herdsman to traverse the border in search of pasture and water for their animals enhancing spreading of goat pox. Following the ineffectiveness of this control measure, the Vietnam Ministry of Agriculture and Rural Development issued free attenuated goat pox vaccine (NAVETCO, Ho Chi Minh City, Vietnam) to the farmers. This has successfully led to effective stamping out of the outbreak without any reported cases until now.

Among important factors in achieving an effective control and preventive measures of a disease is knowing the actual agent and its origin. This is especially so for a country like Vietnam which is in the act of developing goat farming into an industry free of devastating disease like goat pox. The utilization of Vero cell for isolating virus are widely applied for numerous other viruses that are capable of producing cytopathic effect on Vero cell with Capripoxviruses as no exception. Although Vero cell is not recommended for primary propagation of Capripoxviruses [16], it still remains as a preferred precise option which through many other study employing its usage [8, 23, 24].

A comparable appearance of CPE as seen in our study albeit appearing earlier (i.e. at 3 days PI) has been documented [24]. This discrepancy could be accounted for the virulence of the isolated virus used from infected goats between the two studies. However, the common features of cell membrane fusion forming clumped nuclear chromatin were absolutely similar in both studies. The successful adaptation of Capripoxviruses to Vero cell has been previously demonstrated [8, 23] although OIE [18] prefers the usage kid or lamb primary cell culture. There is no objection on the usage of the mentioned primary cultures [18] but this requires sacrificing an animal and these cell cultures are much more difficult to maintain. On the other hand, the continuous cell lines such as Vero are easier to store and maintain under basic laboratory condition and is successful in the isolation of Capripox as seen in this study.

Owing to the difficulty in differentiating goat pox from sheep pox based on clinical signs and serology [25, 26], the conserved structural protein, P32 is used. As employed in this study, the P32 has successfully been used for explicit detection and differentiation of goat pox virus [10, 27–29]. The P32 gene obtained from the samples showed similar resemblance to reference Capripox strains and other goat pox strains [12, 14, 26, 30, 31]. Likewise, the nucleotide analysis also yielded a similarity score to other goatpox virus strains [10, 27]. In addition, the isolated Vietnam goat pox virus is much closely related to LSDV than SPPV. In the amino acid sequence, the main positions that contributed to differentiation of goat pox virus to other Capripox (SPPV and LSDV) are 25, 95, 138, 292 and 325.

The highly conserved P32 gene that encodes for Capripoxviruses envelope was chosen in this study for amino acid and nucleotide analysis that has enabled differentiation of genetic relatedness between goat pox virus and other Capripoxviruses. Nevertheless, a better comparison will be meaningful if the other two highly conserved genes namely GPCR and RPO30 are also incorporated owing to the huge genome size of Capripoxviruses. Undeniably, host specificity of the capripoviruses is not guaranteed even by using those genes [12]. In addition, abundant studies used only P32 gene for epidemiological comparison between the strains [10, 11].

The in vivo and in vitro role of singular or recombinant genes of goat pox virus has been studied. Recombined plasmids of A27, L1, A33 and B5 genes cloned into BHK_21_ triggered humoral and cellular responses in mice and goats [30]. Furthermore, a high specificity and sensitivity by PCR confirmation of P32 recombinant plasmid cloned into Pichia host GSII5 compared to serum neutralization test or indirect ELISA has been recorded [30]. Cloning of F33L and truncated P32 genes by Kumar et al. [31] reported that the purified recombinant was potentially useful for serodiagnosis of capripoxviruses. Utilization of A32L gene from field isolated goat pox virus strain in Taiwan [12] suggested that multiplex PCR and high resolution melt analysis (HRMA) could play and important role in goat pox virus diagnosis. However, there are various choices of host cells for fragment cloning such as eukaryotic [32–34] and prokaryotic cells [11, 34–36]. In the present study, *Escherichia coli* strain DH5α was employed which previously had been successfully used for purification of A32L gene [12].

Although the genetic relationship of studied goat pox virus strain to references have been well depicted on the phylogenetic trees, analyzing sequences provides incredibly interesting insights. First of all, the southward movement of pathogen can be detected via analysis of the decade long constructed goat pox epidemiologic data by Chinese researchers [33, 34, 36]. Together with the high compatibility to our alignment of nucleotides, this proves to the supposition of the origin of the circulating virus [11, 13]. Moreover, geographical analysis revealed that the residue of lysin at position 46 of present isolate contributed to the earlier suggested hypothesis that this is the “signature” of goat pox prevalent in southern Asia [6]. Moreover, the replacement of asparagine at this position was defined among LSDV, SPPV and goat pox virus circulating in middle east [6]. These above observations suggested that the divergence might have resulted from the adaptation of viruses to particular eco-geographic condition.

The P32 gene among various fragment have become the most prevalent gene for Capripoxviruses identification and epidemiological studies. Undoubtedly, exhaustive documentation of researches in analysis has helped to differentiate the virus at genera level and provide a precisely reliable epidemiological data [6, 29]. The absence of aspartic acid at position 55 of P32 gene could be used for differentiation of SPPV to other Capripox genus [10, 36–38]. Apart from three defined “unique characteristic residues” at position of 26, 48 and 95 [39], we found that goat pox can be distinguished from LSDV and SPPV at three other locations including Y138H, M292I and V325I. Our results were in agreement an earlier study [40] when comparing against virus generated from field and vaccine strains. The analysis revealed the changes of six protein resulted from the difference of genomic shift. The authors suggested that the host-specific species and virulence of the virus could be the consequence of those mentioned genomic change during viral attenuation [40, 41].

However, goat pox are known to regulate cell-mediated immune response with various potential insertion sites for other humoral trigger leading to the development of Capripoxvirus-vectors live vaccine [33, 34, 42, 43]. The application of important DNA fragments into recombinant plasmid have been proven to induce similar humoral and cellular immune response with less pathology compared to whole-cell virus vaccine [30, 32, 33].

The essential importance of viral attachment protein is well documented which is also proven to be much more sensitive than ELISA [17]. The homologue of P32 envelope protein of capripox virus to H3L protein of Vaccinia virus [26] has defined similarities. In addition, the sheep pox virus DNA fragment contained ORF that is homologus to the five genes, namely; J6R, H1L, H2R, H3L and H4L of the Vaccinia virus [40–42]. Studies on poxvirus cell entry found that H3 region was the most conserved among four viral mediate MV (D8, A26, A27 and H3) attachment proteins [44]. Consequently, the role of pox virus attachment proteins in initiating the cascade signal and destabilize the plasma membrane leading to pore formation and penetration was reaffirmed [42–44].

## Conclusion

The isolated virus P32 gene sequence from this study has been deposited to the GenBank (Accession No. MN317561). Further analysis sequence of P32 gene fragment revealed highly invariable sites compared to reference sequences accounting for 95.76% and 95.6–99.3% similarities of nucleotide and amino acid sequences, respectively. The phylogram constructed showed three distinct groups of Capripox family with high branching bootstrap values (99.6 and 93.7% of nucleotide and amino acid sequence numbers, respectively). The cluster result suggests a close relationship of Vietnamese goat pox viruses strain to currently circulating strains in Asia.

Even though highly similar, variant gene sites should be investigated to determine their impact. Since only the P32 gene was targeted in the present study, a complete genome of goat pox should be sequenced to enhance a platform for differentiation, diagnosis as well as vaccine production.

## Data Availability

The datasets generated and/or used during the current study are not available to public as it is owned by the Vietnam National University of Agriculture, Vietnam. However, these can be requested via email from the corresponding authors; Dr. Pham Hong Trang (htrang2910@gmail.com) and/or Prof. Dr. Mustapha M Noordin (noordinmm@upm.edu.my).

## Abbreviations

bp: base pair
PBS: phosphate buffered saline
PCR: polymerase chain reaction
GPCR: G-protein-coupled chemokine-receptor
RPO30: 30 kDa DNA-dependent RNA polymerase subunit
OIE: World Organisation for Animal Health
CPE: cytopathic effect
PAGE: polyacrylamide gel electrophoresis
DPI: days post-infection
SPPV: sheep pox virus
LSDV: lumpy skin disease virus
